# A rare case of acute lymphoblastic leukaemia with hemophilia A

**DOI:** 10.1186/1824-7288-35-40

**Published:** 2009-12-11

**Authors:** Rahul Sinha, Kirandeep Sodhi, Biju John, Daljit Singh

**Affiliations:** 1 Department of pediatrics, Command Hospital Air force, Bangalore, Karnataka, India

## Abstract

A rare case of Acute lymphoblastic leukemia with hemophillia in a 12 year old boy is presented in the article. Patient was known case of hemophillia (factor VIII deficiency). He was diagnosed as a case of ALL based on bone marrow examination and immunophenotypic study. Patient was treated as per Children Cancer group guidelines. The main aim of reporting this rare association lies in developing treatment strategies in preventing life threatening bleeding due to this rare association which though may be accidental but need further research.

## Introduction

Acute lympphoblastic leukaemia accounts for around 77% of childhood leukemia. Hemophilia is a congenital bleeding disorder characterized by deficiency of coagulation factor VIII or IX out of which 85% is due to factor VIII deficiency and 15% due to factor IX deficiency. The coincidence of these two diseases together is rare which has led to challenges in developing treatment strategies.

## Case Report

12 yr old boy known case of hemophilia (factor VIII deficiency) diagnosed at 6 month of age presented with intermittent fever (on and off) of 2 month without chills and rigors, significant weight loss of 3 kilograms in 1 one month, generalized weakness of 1 month and joint swelling right knee of 7 days duration. The swelling was spontaneous associated with restriction of movements. There was no family history of malignancy or bleeding disorders.

On examination, the anthropometric measurements were appropriate for age. There was pallor, petechiae and ecchymoses on his legs, along with gingival bleeding, and haematoma on right knee. Systemic examination revealed hepatosplenomegaly. Peripheral blood counts revealed hemoglobin levels of 7.6 g%, white cell counts of 41 × 10^9^/L (blasts 85%), and platelet counts of 100 × 10^9^/L. After receiving 40 U/kg FVIII concentrates, bone marrow aspiration was performed, which showed lymphoblast of 85%. His immunophenotype consisted of CD3 87%, CD7 99%, CD8 10%, CD5 3%, CD10 98%, CD19 18%, CD22 20% and HLA DR negative The cells were negative with MPO staining. RT-PCR for BCR abl negative. A hepatitis viruses screening test found the patient to be hepatitis B and C negative. HIV serological test was negative and cytogenetic test revealed 46, XY. Coagulation screening showed a prolonged activated partial thromboplastin time (APTT) (test 46 seconds, control 28 seconds), with normal prothrombin time. The ultrasound scan of abdomen revealed hepatosplenomegaly, Echocardiography and Xray chest was normal.

After admission to our center, the patient received 30 U/kg of factor VIII. The swelling and pain in his right knee subsided. The patient was placed on induction therapy on the second day, which included oral prednisolone, intrathecal methotrexate, vincristine, daunorubicin. There was no CNS involvement. He was given treatment as per CCG guidelines(Children Cancer Group) which includes 4 weeks of induction, 8 weeks of consolidation, 8 weeks of interim maintenance cycle I (8 weeks), delayed intensification I (8 weeks), delayed intensification II (8 weeks) and maintenance period of 12 weeks. The patient was given intrathecal methotrexate under the cover of factor VIII. During the induction phase there was period of febrile neutropenia, thrombocytopenia and swelling of right knee joint which was managed with antibiotics, platelet transfusion and factor VIII. His peripheral blood counts gradually returned to normal during the treatment, and the patient did not have bleeding complications subsequently. After twenty-eight days of treatment, the patient achieved complete remission. The bone marrow done revealed blasts cells of less than 5% which was consistent with remission. He was discharged from the hospital on the 31st day of induction therapy. He has remained in good condition since then.

## Discussion

In literature, 11 cases of hemophilia with acute leukemia have been documented [1-5] with only 5 pediatric patients. Other authors have reported an increased risk of lymphoma in hemophilia patients, mainly among HIV positive patients [6,7]. Further it has also been observed that hemophiliac patients have long-term abnormalities of immune function from factor concentrate usage [8-10]. Thus, probable factor contributing to a high risk of lymphocytic malignancy could be immunological dysfunction caused by factor concentrate use and/or HIV. However, as of now, only 5 hemophilia patients with acute non-lymphocytic leukemia have been described. It is likely that the association of these two disorders is merely accidental, although further confirmation is needed to bring about more facts under the scanner.

The coincidence of leukemia and hemophilia led to a challenge in developing a treatment strategy. Thrombocytopenia, a complication associated with chemotherapy, can exacerbate bleeding diathesis in severe hemophilia patients and may result in life-threatening bleeding, therefore the threshold for platelet transfusion is kept high. The use of blood products including plasma-derived Factor VIII and platelets may be associated with a risk of virus transmission, in addition to increasing the cost of treatment, leading to a financial burden on the patient and his family.

## Conclusion

The coincidence of hemophilia and leukemia is rare, more so in pediatric age group. This is the sixth case in the literature, which is required to be mentioned for its rarity. The mechanism leading to the combination of these diseases needs further investigation. While both hemophilia and leukemia are severe blood diseases, proper treatment can bring about favorable results.

## Consent

Written informed consent was obtained from the patient for publication of this case report and accompanying images.

## Competing interests

The authors declare that they have no competing interests.

## Authors' contributions

RS: Manuscript preparation. KS: Evaluation and editing of the manuscript. BJ: Evaluation and editing of the manuscript. DS: Evaluation and editing of the manuscript. All authors read and approved the final manuscript.
